# Variation in the prevalence of different forms of bullying victimisation among adolescents and their associations with family, peer and school connectedness: a population-based study in 40 lower and middle income to high-income countries (LMIC-HICs)

**DOI:** 10.1007/s40653-022-00451-8

**Published:** 2022-04-20

**Authors:** Tuhin Biswas, Hannah J. Thomas, James G. Scott, Kerim Munir, Janeen Baxter, M. Mamun Huda, Andre M.N. Renzaho, Donna Cross, Helal Uddin Ahmed, Rashidul Alam Mahumud, Abdullah A Mamun

**Affiliations:** 1 Institute for Social Science Research, The University of Queensland, Brisbane, Australia; 2 ARC Centre of Excellence for Children and Families over the Life Course, The University of Queensland, Brisbane, Australia; 3 QIMR Berghofer Medical Research Institute, Herston, QLD Australia; 4 Metro North Mental Health, Royal Brisbane and Women’s Hospital, Herston, Brisbane, QLD Australia; 5 School of Public Health, Faculty of Medicine, The University of Queensland, Herston, QLD Australia; 6 Queensland Centre for Mental Health Research, Wacol, Brisbane, QLD Australia; 7 Developmental Medicine Center, Boston Children’s Hospital, Harvard Medical School, Boston, MA USA; 8 School of Social Sciences and Psychology, Western Sydney University, 2751 Penrith, NSW Australia; 9 Telethon Kids Institute, The University of Western Australia, West Perth, Australia; 10 Child Adolescent and Family Psychiatry, National Institute of Mental Health (NIMH), Dhaka, Bangladesh; 11 School of Social Sciences, Western Sydney University, 2751 Penrith, Australia; 12 Translational Health Research Institute, Western Sydney University, 2751 Penrith, Australia; 13 Centre for Health Research, University of Southern Queensland, 4350 Toowoomba, QLD Australia; 14 Institute for Social Science Research, The University of Queensland, 80 Meiers Road, 4068 Indooroopilly, Queensland Australia

**Keywords:** Traditional bullying, Cyberbullying, Family functioning, Peer relationships and school connectedness

## Abstract

This study aimed to estimate the prevalence of different forms of bullying victimization experiences and their association with family functioning, peer relationships and school connectedness among adolescents across 40 lower and middle income to high-income countries (LMIC-HICs). Data were drawn from the Health Behaviour in School-Aged Children (HBSC) school-based survey of adolescents aged 11–15 years, between 2013 and 2014. We estimated the weighted prevalence by categorising experiences into traditional bullying victimization only, cyberbullying victimization only, and combined traditional and cyberbullying victimization, at country and country income classification. We used multinominal logistic regression models to estimate the adjusted association with the form of bullying victimization by demographic characteristics, family functioning, peer relationships and school connectedness. Overall, 8.0% reported traditional bullying victimization only (8.8% males, 7.4% females), 2.3% of adolescents reported cyberbullying victimization only (2.1% males, 2.2% females), and 1.7% reported combined traditional and cyber bullying victimization (1.7% males, 1.8% females). All three forms of bullying victimization during adolescence were significantly associated with poor family functioning, poor peer relations and poor school connectedness. A consistent finding is that traditional bullying victimization is considerably more common among adolescents across both LMICs and HICs than cyberbullying victimization. This study also demonstrated that a significant proportion of adolescent’s experience victimization in both forms. Positive family functioning, strong peer relationships and greater school connectedness are associated with a lower risk of both forms of bullying victimization.

**Supplementary information:**

The online version contains supplementary material available at 10.1007/s40653-022-00451-8.

## Introduction

Bullying victimization is a serious global public health problem among adolescents and is associated with increased risk of adverse physical, cognitive, and mental health outcome (Biswas, Scott, Munir, Thomas, et al., 2020; Przybylski & Bowes, 2017). There is a vast amount of evidence linking bullying experiences to development of emotional, cognitive, social, and behavioural problems (Le et al., 2019). The significant psychosocial harms from bullying among adolescents create major challenges for mental health promotion programs and services in schools (Smith & Slonje, 2010). Traditional bullying typically occurs face-to-face and includes physical, verbal and relational forms of interaction (Smith & Slonje, 2010). Cyberbullying is an extension of this behaviour in online environments enacted through the use of electronic communications, such as e-mail, instant or text messaging, or social networking posts, in real time or asynchronously (Slonje & Smith, 2008). Irrespective of the mode of behaviour, bullying is defined as actions that are intentional, repeated and involve a power imbalance to the extent that it makes it difficult for the victim to defend themselves (Gladden, 2014; Thomas et al., 2019). There is now significant interest in understanding the level of co-occurrence of bullying victimization across contexts, especially given the wide uptake of electronic communication tools among youth. (Modecki et al., 2014). Recently, social media use among adolescents living in high income settings has become the primary form of communication with doubling of internet use in the past decade (Britain, 2013; Kelly et al., 2018). Concerns have been raised that cyberbullying victimization may lead to greater harm than traditional bullying victimization since it can have an ever pervasive presence in the victims’ homes and interfere in all aspects of their daily lives (Patchin & Hinduja, 2018; Sticca & Perren, 2013). However, this question has not been tested empirically in large population-based samples. Both the individual and cumulative effects of multi-model bullying victimization experiences are associated with poorer outcomes for victims (Thomas et al., 2017). Since traditional and cyberbullying victimization experiences often co-occur, studies examining prevalence should disaggregate this group from those who experience cyberbullying alone (Jadambaa et al., 2019; Waasdorp & Bradshaw, 2015). This is especially important when trying to tease apart the independent effects of different types of bullying victimization exposure.

Due to the absence of a standardised definition, validated cut-offs, and nationally representative samples, the global prevalence of cyberbullying has been challenging to estimate (Zhang et al., 2018). A large population study in England in 2014-15 reported that 36% of girls, and 24% of boys experience some form cyberbullying victimization “at least 2–3 times a month” in the previous two months (Bonanno & Hymel, 2013). Data from the US National Crime victimization Survey School Crime Supplement shows that in 2015 approximately 16% of students aged 12–18 years reported cyberbullying victimization (Elledge et al., 2013). However, the overlap between cyberbullying and traditional bullying victimization experiences among youth is much less documented, likely because historically there have been few measures of both traditional and cyber forms of bullying victimization (Thomas et al., 2015, 2017, 2019). Most previous cross-national studies or systematic reviews on prevalence are limited to research that examined traditional forms of bullying victimization only or the study’s measurement approach does not enable separate prevalence estimates for different types of bullying victimization exposure (Craig et al., 2009; Pernille Due & Bjørn Evald Holstein, 2008; Jadambaa et al., 2019). Another previous study of prevalence examined the overlap between traditional and cyberbullying bullying, though this study did not examine prevalence by geography, and largely included data from high-income countries (Modecki et al., 2014).

Previous research has identified a range of risk factors for bullying victimization including sociodemographic characteristics and a prior history of traditional and cyberbullying victimization (Baldry et al., 2015; Kowalski et al., 2014; Paez, 2018; Patchin & Hinduja, 2011; Pelfrey Jr & Weber, 2013; Rice et al., 2015; Tanrikulu & Campbell, 2015). Some studies have reported sex differences in prevalence estimates, with more females than males experiencing cyberbullying victimization (Elgar et al., 2019). A longitudinal study in Australia among the adolescents aged 13-years found students who had social and emotional difficulties were more likely to be victims of both cyberbullying and traditionally bullying, than those experienced traditionally bullying only (Cross et al., 2015). This relationship is likely bidirectional, with cross-sectional research showing that exposure to either cyberbullying or traditionally bullying increases concurrent risk of mental health difficulties (Yang et al., 2021). It is also well-established that peer and family factors can influence the risk of bullying victimization (Biswas, Scott, Munir, Thomas, et al., 2020). A lack of parental involvement, family support, poor school connectedness (Duggins et al., 2016), previous experiences of traditional bullying and school environments where bullying behaviour is normalized have also been identified as predictors of cyberbullying victimization (Arsenio & Gold, 2006; Pernille Due & Bjorn Evald Holstein, 2008; Roberson & Renshaw 2018; Thomas et al., 2017). Different studies suggest that adolescent bullying might be more closely related to income inequality than to socioeconomic status (Elgar et al., 2009; Kawachi & Kennedy; Kennedy 2002). In addition, studies also found that early-life exposure to income inequality is associated with an increased risk of being bullied in adolescence (Cantone et al., 2015; Torsheim et al., 2004). However, country specific income inequality has not been explored.

There are significant knowledge gaps relating to bullying victimization in adolescents. Most research has not disaggregated cyberbullying only from combined traditional and cyberbullying victimization with the latter more strongly associated with poor mental health in adolescents (Wang et al., 2019). Using this classification of bullying victimization experiences, prevalence and associations between sex and income inequality in LMICs are yet to be examined. Furthermore, no study to-date, has evaluated the association between cyberbullying victimization, traditional bullying victimization and combined traditional and cyberbullying victimization and family functioning, peer relationships and school connectedness across a wide distribution of countries with varying income levels and cultures.

The current study aimed to address these gaps by providing the most comprehensive overview to-date of the variation in the prevalence of traditional bullying victimization, cyberbullying victimization, and combined traditional and cyberbullying victimization, and to examine their independent association with indices of economic development, family functioning, peer relationships and school connectedness in 40 LMIC-HICs.

### Methods

#### Data sources

Data were obtained from the World Health Organization (WHO) Health Behavior in School-Aged Children (HBSC) study in 2013/2014 undertaken in 40 HIC-LMICs (*N* = 214,080). The HBSC study is a cross-national representative (Roberts et al., 2009) school-based survey into the health and well-being of adolescents with data collected through self-completion questionnaires administered in the classrooms of over 40 countries in Europe and North America. The HBSC is conducted in collaboration with the WHO Regional Office for Europe. The questionnaire is administered to adolescents aged 11–15 years and captures information on a wide range of health indicators using validated items from ten core modules including: nutrition, physical activity, hygiene, mental health, alcohol use, tobacco use, drug use, sexual behaviors, violence/injury, and protective factors. Samples were drawn using cluster sampling, with school classes or the whole school as the primary sampling unit. The study design and selection procedure of the participants are similar across the participating HBSC countries. The detail of the HBSC study design has been described elsewhere (Roberson & Renshaw, 2018).

### Measurements

**Traditional bullying victimization.** Respondents were asked to read a short definition of bullying which incorporated power imbalance and the intent to harm and distinguished the behaviour from teasing. Adolescents were then asked “How often have you been bullied in school in the past couple of months?” Students were given five response options, ranging from *never* to *several times a week*. Possible response options were “I have not been bullied at school in the past couple of months”, “It has happened once or twice”, “2 or 3 times a month”, “About once a week” and “Several times a week”. In this study, the responses were dichotomized, such that those bullied “2 or 3 times a month” or more often were coded as having experienced traditional bullying victimization.”, for the reasons that adolescents were presented with a definition of bullying prior to questions (Roberson & Renshaw, 2018), and is consistent with a previous cross-national study of bullying from an earlier HBSC survey (Pernille Due & Bjorn Evald Holstein, 2008).

**Cyberbullying victimization.** Respondents were asked to read a short definition of cyber bullying victimization. Adolescents were then asked “In the past couple of months how often have you been cyberbullied (e.g., someone sent mean instant messages, email or text messages about you; wall postings; created a website making fun of you; posted unflattering or inappropriate pictures of you online without permission or shared them with others)? Response options included ‘I have not been cyberbullied in the past couple of months’, ‘It has happened once or twice’, ‘2 or 3 times a month’, ‘once per week’, and ‘several times per week. Responses were dichotomized whereby those who endorsed the response of “2 or 3 times a month” or more often in the past couple of months were categorized as having experienced cyberbullying victimization.

***Combined traditional and cyberbullying victimization.*** Adolescents who endorsed both traditional bullying and cyberbullying items were categorized as having experienced combined traditional and cyberbullying victimization.

**Socioeconomic status.** The socioeconomic status of the participant’s family was a composite of three indicators: “Does your family have a car or a van?” [‘No’ (0), ‘Yes’ (1), ‘Yes, two or more’ (2)]; “Do you have your own bedroom?” [‘No’(0)]; ‘Yes’ (1)], and “During the past year, how many times did you travel away on holiday (vacation) with your family?” [‘Not at all’ (0), ‘Once’ (1), ‘Twice’ (2), ‘More than twice’ (3)]. These indicators were summed to produce a composite score of family socioeconomic position, ranging from 0 (lowest affluence) to 6 (highest affluence) (Torsheim et al., 2004).

**Family functioning.** Family functioning was captured through a composite measure of ten items measured on a five-point Likert scale: ‘Strongly agree’, ‘Agree’, ‘Neither agree/disagree’, ‘Disagree’, or ‘Strongly disagree’. The items included the following item stems: “In my family- (i) “I think the important things are talked about”; (ii) “When I speak, someone listens to what I say” (iii) ***“***We ask questions when we don’t understand each other”; (iv) When there is a misunderstanding we talk it over until it is clear”; (v) “My family really tries to help me”; (vi) “I get the emotional help and support I need from my family”; (vii) “I can talk about my problems with my family”; (viii) “My family is willing to help me make decisions”; (ix and x) “How easy is it for you to talk to your Mother/Father about things that really bother you.

**Peer relationships.** A measure of the quality of peer relationships was captured through three items measured on a five-point Likert scale: ‘Strongly agree’, ‘Agree’, ‘Neither agree/disagree’, ‘Disagree’, or ‘Strongly disagree’. The items included the following item stems: (i) “The students in my class(es) enjoy being together”; (ii) “Most of the students in my class(es) are kind and helpful”; (iii) “Other students accept me as I am”.

**School connectedness.** School connectedness was captured through three items measured on a five-point Likert scale: ‘Not at all, ‘A little’, ‘Some’, ‘Disagree’, ‘A lot’. The items included the following item stems: (i) “I feel that my teachers accept me as I am”; (ii) “I feel that my teachers care about me as a person”; (iii) I feel a lot of trust in my teachers. Possible response options to each of these questions were ‘Strongly agree’, ‘Agree’, ‘Neither agree/disagree’, ‘Disagree’, ‘Strongly disagree’. Two additional items included (iv) “How do you feel about school at present?”; and (v) “How pressured do you feel by the schoolwork you have to do?”

**Country’s development indices.** We included the following country specific national indices in our adjusted model. (i) Gross Domestic Product (GDP) i.e., total value of goods produced and services provided; (ii) Human Development Index (HDI) i.e., dimensions of human development including life expectancy, standard of living measured by Gross National Income and per capita income adjusted for price level; (iii) Gender Development Index (GDI) i.e., gender gap in human development accounting for disparities between women and men in the basic HDI dimensions; and (iv) Gini Index i.e., measure of economic inequality and income distribution. We used country specific GDP, HDI, GDI, and Gini Index corresponding to the survey year as reported by the World Bank. For a few countries, Index Mundi data were used where GDP and Government expenditure on education were not listed in the World Bank list.

## Statistical analyses

First, we derived composite measure of the family functioning, peer relationships and school connectedness based on the associated items mentioned above. Composite score for family functioning, peer relationships and school connectedness were estimated using factor analysis. The variables used in this analysis were standardized (mean zero and variable one) and principal-component analysis was used to extract the factors. Only the first factor was considered, and corresponding factor scores were used to construct the score for family functioning, peer relationship and school connectedness. The family functioning, peer relationships and school connectedness variables were further categorised into tertiles (low, medium and high) based on the percentile distribution of the scores.

Secondly, weighted prevalence of traditional bullying victimization, cyberbullying victimization, and combined traditional and cyber bullying victimization (with 95% CIs) were calculated by country, sex as well as family and school level factors in this study. The data were weighted by sampling weight to make the estimate nationally representative.

Finally, we conducted multinomial logistic regression analysis to examine the factors associated with traditional bullying victimization, cyberbullying victimization and combined traditional and cyberbullying victimization. First, we conducted unadjusted multinomial logistic regression to select variables, which had a bivariate association with traditional bullying victimization, cyberbullying victimization, and combined traditional and cyberbullying victimization. We then fitted final multinomial logistic regression models adjusted by age, sex, socioeconomic position, family functioning, peer relationships and school connectedness, survey year, as well as country level variables- Gross domestic product (GDP), measure of income inequality (Gini), Gross domestic income (GDI) and Human development index (HDI) to explore independent factors associated with traditional bullying victimization, cyberbullying victimization, and combined traditional and cyberbullying victimization .

## Results

**Fig. 1:**
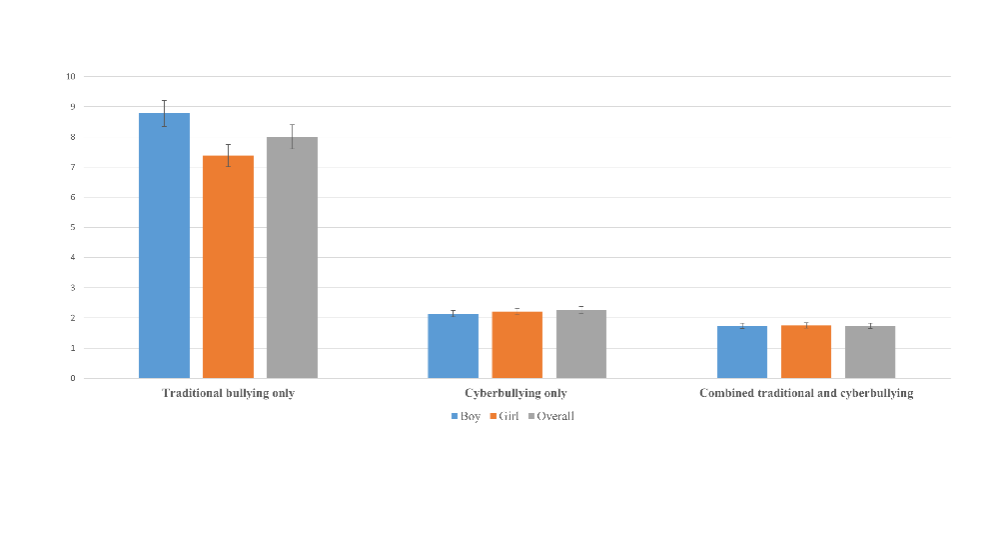
Overall prevalence of different types of bullying victimization by gender

**Fig. 2:**
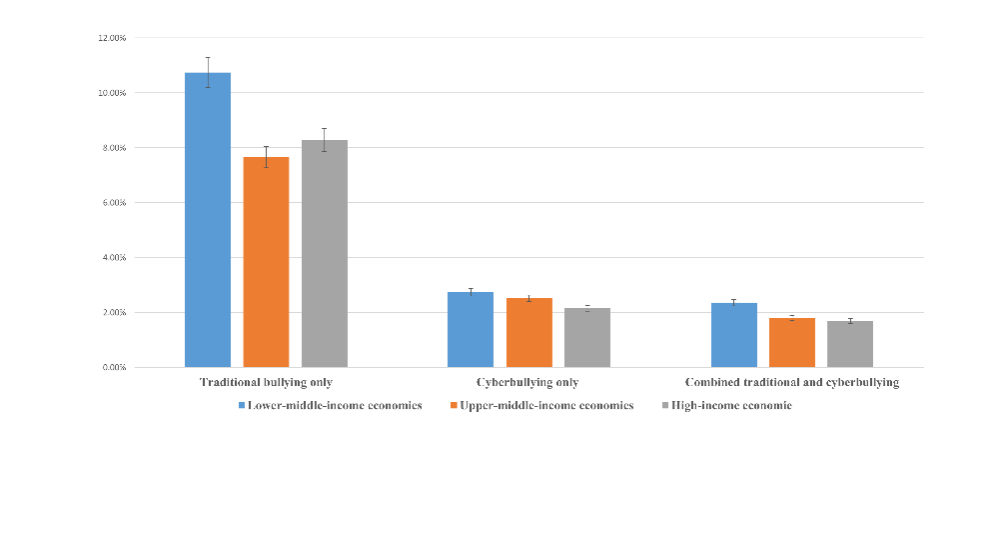
Overall prevalence of different types of bullying victimization by economic positions

Of the 214,080 adolescents aged 11–15 years, the mean age was 13.5 years (SD 1.62), 49.24% were male (*N* = 105,414) and 50.76% were female (108,666). Overall, 8.0% of adolescents reported traditional bullying victimization only (males: 8.8% and females: 7.4%), 2.3% of exposure to cyberbullying only (females: 7.0% and males: 5.0%), while and 1.7% reported combined traditional and cyberbullying victimization (males: 2.1% and females: 2.2%) (Figure-1). According to the country income classification, pooled prevalence of cyberbullying only was the least prevalent form of victimization, with it being slightly higher in LMICs (2.74%, 2.42–3.09%), compared to upper-middle-income (2.52%, 2.32–2.73%), and HICs (2.15%, 2.08–2.23%), but not significantly so. By comparison, traditional bullying victimization and combined traditional and cyberbullying victimization were more common forms of victimization in all countries and were particularly prevalent in LMICs (traditional bullying victimization: 10.7%, 10.1–11.4%; combined traditional and cyberbullying victimization: 2.4%, 2.0-2.7% Fig. 2). The highest prevalence figures of cyberbullying victimization were observed in Republic of Moldova (5.0%) and lowest in Germany (1.0%). The highest prevalence of traditional bullying victimization was observed in Italy (19.0%) and lowest in Austria (1·5%). The highest prevalence of combined traditional and cyberbullying victimization (supplementary Table 1) was observed in Republic of Moldova (4.2%) and lowest in Austria (1.0%).

Table 1 shows the prevalence of traditional bullying victimization, cyberbullying victimization, and combined traditional and cyberbullying victimization by age group, socioeconomic status, family functioning, peer relationships and school connectedness. The prevalence of cyberbullying victimization increased with age and reductions in SES. Almost without exception, there was a lower prevalence of cyberbullying victimization, traditional bullying victimization and combined traditional and cyberbullying victimization among adolescents, who reported better family functioning, peer relationships and school connectedness (Table 1).

**Table 1:** Prevalence by demographic characteristics

Sociodemographic status	Traditional bullying only	Cyberbullying only	Combined traditional and cyberbullying
**Age group**			
11 years	9.8 (9.6–10.0)	2.0 (1.9–2.1)	1.7 (1.6–1.8)
13 years	8.6 (8.4–8.8)	2.3 (2.2–2.4)	2.0 (1.9–2.1)
15 years	5.9 (5.7–6.1)	2.3 (2.2–2.4)	1.6 (1.5–1.7)
**Socioeconomic Position**			
0	10.29 (9.53–11.1)	2.49 (2.12–2.92)	3.48 (3.04–3.99)
1	10.45 (10.01–10.91)	2.39 (2.18–2.62)	1.97 (1.78–2.19)
2	8.67 (8.38–8.97)	2.25 (2.1–2.42)	1.76 (1.63–1.91)
3	8.18 (7.93–8.43)	2.05 (1.93–2.18)	1.65 (1.54–1.77)
4	7.19 (6.95–7.44)	1.92 (1.79–2.05)	1.43 (1.32–1.55)
5	7.77 (7.47–8.08)	2.11 (1.95–2.28)	1.68 (1.54–1.83)
6	7.36 (7.01–7.73)	2.46 (2.25–2.68)	1.84 (1.67–2.04)
**Family functioning**			
Low	10.97 (10.72–11.23)	3.15 (3.01–3.3)	2.76 (2.63–2.9)
Medium	7.66 (7.45–7.88)	1.72 (1.62–1.83)	1.19 (1.1–1.28)
High	5.45 (5.27–5.64)	1.27 (1.18–1.36)	0.88 (0.8–0.96)
**Peer relationship**			
Low	14.18 (13.91–14.45)	2.87 (2.74-3)	3.3 (3.17–3.44)
Medium	6.57 (6.38–6.77)	2.02 (1.91–2.13)	1.23 (1.15–1.32)
High	4.43 (4.29–4.58)	1.69 (1.6–1.78)	0.82 (0.76–0.89)
**School connectedness**			
Low	10.47 (10.24–10.71)	3.01 (2.88–3.14)	2.75 (2.63–2.88)
Medium	7.65 (7.45–7.85)	1.9 (1.8–2.01)	1.36 (1.27–1.45)
High	6.3 (6.12–6.49)	1.48 (1.39–1.57)	1 (0.93–1.08)

Meta-regression analyses showed a positive association between the prevalence of cyberbullying victimization (β = 1.33, 95% CI: 1.00-1.68%, p < 0.001), traditional bullying victimization (β = 1.70, 95% CI: 1.48-1.92%, p < 0.001) and combined traditional and cyberbullying victimization (β = 2.09, 95% CI: 1.82-2.36%, p < 0.001) and country gender development Index (Supplementary Figs. 1–3). However, the prevalence of traditional bullying victimization, cyberbullying victimization, and combined traditional and cyberbullying victimization was not associated with country GDP, Gini and Human development index.

Table 2 shows the multinominal logistic regression (adjusted model) of cyberbullying victimization, traditional bullying victimization and combined traditional and cyberbullying victimization for the overall sample. Females were significantly more likely than males to be victimized online (cyberbullying victimization: RRR: 1.10, 95% CI: 1.00-1.11%) and less likely to experience traditional bullying only (RRR 0.94, 95% CI 0.87%-1.02%) and combined cyberbullying and traditional bullying victimization (RRR: 0.76, 95% CI: 0.73-0.79%). Those adolescents who had poorer family functioning had significantly higher rates of traditional bullying victimization (RRR: 1.77, 95% CI: 1.69-1.87%), cyberbullying victimization (RRR: 2.33, 95% CI: 2.10-2.58%). Poor family functioning was more strongly associated with those who experienced combined traditional and cyberbullying victimization (RRR: 2.48, 95% CI: 2.21-2.79%). Adolescents who had poorer peer relationships had significantly higher risk of experiencing cyberbullying victimization (RRR: 1.42, 95% CI: 1·29%-1·56%), traditional bullying victimization (RRR: 3.37, 95% CI: 3.20-3.55%) and combined traditional and cyberbullying victimization (RRR: 3.34, 95% CI: 2.97-3.75%). Notably, traditional bullying victimization and combined traditional and cyberbullying victimization were more strongly associated with poor peer relationships compared to cyberbullying only. By contrast, while reduced school connectedness was associated with all forms of victimization, it was more strongly associated with cyberbullying victimization (Cyberbullying victimization only RRR: 1.56, 95% CI: 1·41%-1·74% and combined traditional and cyberbullying victimization RRR: 1.56, 95% CI: 1.38-1.76%) compared to traditional bullying victimization only (RRR: 1.21, 95% CI: 1.15-1.28%).

**Table 2:** Multinominal logistic regression analysis of relative risk relative risk ratio (RRR) of being cyberbullying victimization, traditional bullying victimization and Combined traditional and cyberbullying

Sociodemographic status	Traditional bullying only		Cyberbullying only		Combined traditional and cyberbullying	
**Age group**	**RRR (95% CI)**	**P value**	**RRR (95% CI)**	**P value**	**RRR (95% CI)**	**P value**
11 years	**Ref**	**Ref**	**Ref**	**Ref**	**Ref**	**Ref**
13 years	0.73 (0.7–0.76)	0.00	0.96 (0.87–1.05)	0.35	0.9 (0.82-1)	0.05
15 years	0.43 (0.41–0.45)	0.00	0.81 (0.74–0.9)	< 0.001	0.54 (0.49–0.61)	0.00
**Sex**						
Boy	**Ref**	**Ref**	**Ref**	**Ref**	**Ref**	**Ref**
Girl	0.76 (0.73–0.79)	0.00	1.10 (1.00-1.11)	0.47	0.94 (0.87–1.02)	0.15
**Socioeconomic Position**						
0	1.33 (1.18–1.49)	< 0.001	0.89 (0.71–1.13)	0.34	1.58 (1.28–1.95)	< 0.001
1	1.37 (1.26–1.48)	< 0.001	0.95 (0.81–1.11)	0.49	0.95 (0.8–1.13)	0.60
2	1.1 (1.02–1.18)	< 0.001	0.86 (0.75–0.98)	0.03	0.77 (0.66–0.9)	< 0.001
3	1.03 (0.96–1.11)	0.35	0.78 (0.69–0.89)	< 0.001	0.79 (0.68–0.91)	< 0.001
4	0.93 (0.87-1)	0.07	0.74 (0.65–0.84)	< 0.001	0.72 (0.62–0.84)	< 0.001
5	1.05 (0.97–1.14)	0.20	0.88 (0.77–1.01)	0.07	0.88 (0.75–1.03)	0.11
6	**Ref**	**Ref**	**Ref**	**Ref**	**Ref**	**Ref**
**Family functioning**						
Low	1.77 (1.69–1.87)	0.00	2.33 (2.1–2.58)	< 0.001	2.48 (2.21–2.79)	< 0.001
Medium	1.25 (1.19–1.32)	0.00	1.29 (1.16–1.44)	< 0.001	1.16 (1.02–1.32)	0.02
High	**Ref**	**Ref**	**Ref**	**Ref**	**Ref**	**Ref**
**Peer relationship**						
Low	3.37 (3.2–3.55)	< 0.001	1.42 (1.29–1.56)	< 0.001	3.34 (2.97–3.75)	< 0.001
Medium	1.46 (1.39–1.55)	< 0.001	1.05 (0.95–1.16)	0.35	1.33 (1.16–1.51)	< 0.001
High	**Ref**	**Ref**	**Ref**	**Ref**	**Ref**	**Ref**
**School connectedness**						
Low	1.21 (1.15–1.28)	< 0.001	1.56 (1.41–1.74)	< 0.001	1.56 (1.38–1.76)	< 0.001
Medium	1.02 (0.97–1.08)	0.41	1.11 (1.00-1.24)	0.04	1.05 (0.93–1.19)	0.41
High	**Ref**	**Ref**	**Ref**	**Ref**	**Ref**	**Ref**

## Discussion

The present study based on the WHO HBSC data provides a comprehensive summary of the prevalence and correlates of traditional bullying, cyberbullying, and combined traditional and cyberbullying victimization across 40 HIC-LMICs. This is the first study to examine prevalence in a multi-country meta-analysis using a 3-category prevalence method, which has enabled a more nuanced estimation of prevalence and correlates. There are four key findings from these data. Traditional and combined traditional and cyberbullying victimization are much more common in all countries compared to cyberbullying alone. Second, there was wide variation between countries in the prevalence of different forms of bullying victimization, underscoring the salience of social and cultural factors on bullying victimization. Third, there was a consistent pattern of females being more likely to experience cyberbullying victimization while males were more likely to experience traditional bullying victimization. Finally, problems with family functioning, peer relationships and school connectedness were associated with all forms of bullying victimization.

The higher prevalence of traditional bullying victimization suggests interventions only targeting cyberbullying (Cantone et al., 2015) are inadequate for adolescents. Although it has been suggested that cyberbullying is increasing (Wang et al., 2019), it has been acknowledged that cyberbullying victimization occurs alongside traditional bullying victimization (Sheehan et al., 2017; Thomas et al., 2017). Consistent with other studies, we found that most of the adolescents who experienced cyberbullying victimization also experienced traditional bullying victimization, across LMIC to HIC.

The variation in different forms of bullying victimization prevalence by country, gender as well as development indices by country, provide unique opportunities to examine its social and cultural determinants. For example, highest prevalence of cyberbullying victimization was observed in Republic of Moldova, highest prevalence of traditional bullying victimization was observed in Italy and the highest prevalence of combined traditional and cyberbullying victimization was observed in Republic of Moldova. The variation in prevalence may reflect differences in social and networking usage, as well as implementation of preventive interventions and national policies to reduce cyberbullying victimization. In addition, this study found most of the countries didn’t have repeated follow-up data to monitor the country progress.

In our study we found that females were more likely to experience cyberbullying victimization than males. This builds on the previous meta-analytic studies that have examined the influence of gender and age on involvement in bullying. Smith at al 2019 found that males were more likely to report bullying victimisation, although when a sub-group of adolescents aged 13–15 years were examined, this gender difference became non-significant. This may be explained by the increase in cyberbullying victimization in this age group. Smith et al., 2019T found that cyberbullying victimization was more common among females compared to males, although they did not account for the three-role victimisation classification examined in the current study.

In contrast to the literature on bullying victimization, involvement in bullying perpetration is consistently associated with male gender (Smith et al., 2019). Sun et al(Sun et al., 2016) found that males were more likely to be involved in cyberbullying perpetration behaviours than females. These gender differences are commonly attributed to differential gender socialisation and normative expectations of behaviour for males and females (Felix & Green, 2009). However, it is also likely to be influenced by other socio-cultural factors such as age, type of bullying, country and culture, and the historical period (Smith et al., 2019). In addition, irrespective of gender differences, the measurement approach and the way bullying victimisation and perpetration are measured in large cross-national surveys likely also influences overall prevalence estimation. Multi-dimensional measurement models that capture both the bullying role (victimisation and perpetration) and the type of bullying behaviour (e.g., physical, verbal relational, cyber) show promise in addressing some of the disadvantages of unidimensional measurement models (Thomas et al., 2018).

Bullying victimisation during childhood and adolescence has previously been identified in the Global Burden of Disease Study as a causative risk factor for major depressive disorder and anxiety disorders (Jadambaa et al., 2020). Adolescents, and now particularly girls are a focus of the global health issue for achieving Sustainable Development Goals (SDGs) (Patton et al., 2016; Reiner et al., 2019). Policy makers must make strategic decisions about public health interventions that represent cost-effective investments to reduce risk factors for disease. There is mounting evidence from health economic modelling to suggest that interventions aimed at reducing bullying are cost effective (Jadambaa, 2020). Approximately 50% of adolescents grow up in countries with a high burden of communicable, maternal, nutritional and other adolescent health problems, including exposure to violence (Rivers & Smith, 1994) and many are in LMICs. Over time, as the burden of communicable diseases decreases in LMIC countries, the burden of non-communicable disease (including mental disorders) will increase (Erskine et al., 2015). It is likely there will be greater demand for evidence-based interventions for reducing risk factors for mental disorders. In line with this evidence, our study, identified that cyberbullying was higher in LMICs compared with HICs. For this reason, we argue that interventions aimed at reducing all forms of bullying be developed and adapted for the unique socio-cultural needs of LMIC”.

Given that bullying victimization is associated with peer, family, and school factors, it is important that intervention programs involve a comprehensive anti-bullying approach that considers the social ecology of the individual student. The evidence from high-income countries suggests that bullying interventions are less effective with adolescents compared to primary school aged children (Heymann et al., 2019) and there is limited evidence on anti-bullying interventions for adolescents in low-and middle-income countries (Sivaraman et al., 2019). More research is required to improve intervention gains for adolescents in particular.

In our study country specific analysis, we found that for almost all countries there was an association between all forms of bullying victimization among adolescents and problems with family functioning, peer relations and school connectedness. Broadly, this pattern of findings suggests that the strength of association with key correlates within the family, peer and school domains are similar for both traditional bullying and cyberbullying victimization. It is plausible that these relationships are most likely dose-response dependent, rather than indicative of the type of bullying exposure. Our findings are also consistent with studies from HICs showing that family and parental support reduces bullying victimization (Biswas, Scott, Munir, Renzaho, et al., 2020; Moore et al., 2017). Another study using GSHS data from 83 LMIC-HICs in the six World Bank regions demonstrated that parental involvement in the lives of adolescents and peer support were strongly associated with reduced levels of traditional bullying victimization (Biswas, Scott, Munir, Thomas, et al., 2020). Our study suggests these are also associated with reduced likelihood of cyberbullying victimization.

Despite cultural and demographic differences between countries observed, improving family connection, peer relations and school connectedness for adolescents may be the foundations globally to reducing bullying victimization. In all countries, culturally appropriate interventions to improve the relationships between adolescents and their parents and peers may assist in reducing all forms of bullying victimization as well as reduce other mental health and high-risk behaviours in adolescents.

This study has attempted to provide a comprehensive overview of the available evidence related to cyberbullying traditional bullying and combined traditional bullying and cyberbullying victimization, although it has a number of limitations. Firstly, the HBSC measurement of bullying victimization was self-reported. While self-report is an accepted method there is a limitation of possible shared method variance (Thomas et al., 2015). Second, the study design was cross-sectional, therefore the establishment of causality was not possible. It is also recognised that there are numerous other risk factors associated with bullying victimization, such as internalising problems that were not examined in the current study. While it is well recognising that bullying victimization is associated with increased risk of future internalising problems (Moore et al., 2017), there is also evidence that shows internalising problems confer risk of future bullying victimization (Gámez-Guadix et al., 2013). The study is limited to victimization experiences. Involvement in bullying is dynamic and like traditional and cyber experiences co-occur, perpetration also typically occurs alongside victimization. Additional data is needed to improve the evidence base and modelling approaches to better capture the dynamic and multi-faceted pathways associated with exposure to bullying victimization. It is only possible to examine these pathways in longitudinal designs. Finally, data were collected between 2013 and 2014 present’s differential significant period may effects on prevalence of different types of bullying. However, our multivariable estimates were adjusted for period effects. The study findings are mainly from North American and European countries, with some regions (Asia and Africa) not represented. Maintaining data monitoring in those countries should remain a priority. Although WHO collected some traditional bullying information in Asia and the African region but cyberbullying still not include in the bullying question module. To assess the county progress, it is also important collect the data routinely.

Nonetheless, the study has a number of strengths that help uniquely estimate the prevalence of different types of bullying victimization in adolescence. First, the HBSC methodology represents a collaborative standardised questionnaire. Data collection was standardised and always occurred during a regular class period. The strength current study is that it is a multi-country study that used equivalent bullying measures conducted within the same cross-sectional time period, therefore eliminating measurement and time period differences. Another strength is the use of survey data with large random sample sizes taken from a wide variety of international geographical and cultural settings. Finally, the analyses were inclusive of data from 40 countries, captured within a similar period of time.

The findings of the study confirm that a significant proportion of adolescents experienced both traditional bullying and cyberbullying victimisation over the previous couple of months. The variation between countries and the findings that poor family functioning, peer relationships and school connectedness increase the risk of bullying victimization suggests that the risk of exposure to all forms of bullying victimization is modifiable. Mainly, poor family function means functioning occurs within families with high levels of conflict, disorganization, and poor affective and behavioural control, which negatively impact child and adolescent health risk behaviour. Given that bullying victimization in schools increase the risk of anxiety (Biswas, Scott, Munir, Renzaho, et al., 2020) and depression (Moore et al., 2017), a meaningful reduction in the prevalence of bullying offers an opportunity to reduce the global burden of disease attributable to the most prevalence forms of mental illness. This highlights the importance of family functioning, peer relationships and school connectedness. Our study supports consideration of these intermediate factors in the design of school and community- wide programs that promote social and family engagement.

## Electronic supplementary material

Below is the link to the electronic supplementary material.

Supplementary Material 1
