# Supplementary material for: Variation in the prevalence of different forms of bullying victimisation among adolescents and their associations with family, peer and school connectedness: a population-based study in 40 lower and middle income to high-income countries (LMIC-HICs)
Source: J Child Adolesc Trauma. 2022 Apr 20;15(4):1029–39. doi: 10.1007/s40653-022-00451-8 (PMC9684371; doi:10.1007/s40653-022-00451-8)
Supplement: Supplementary file 1 — Supplementary Material 1 [file 40653_2022_451_MOESM1_ESM.docx]

**
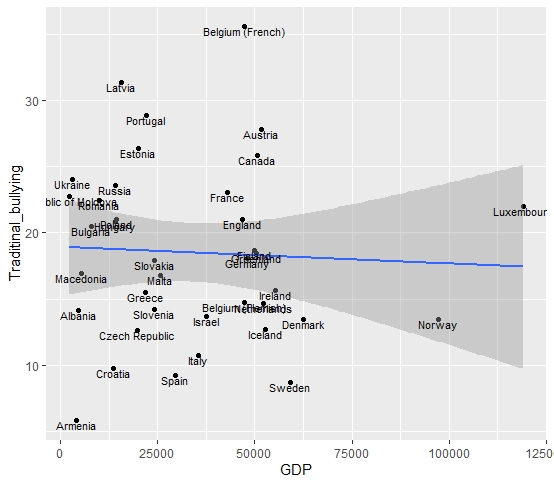

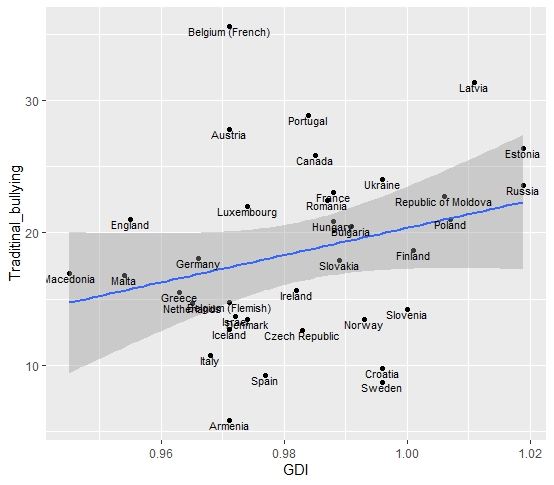
** **
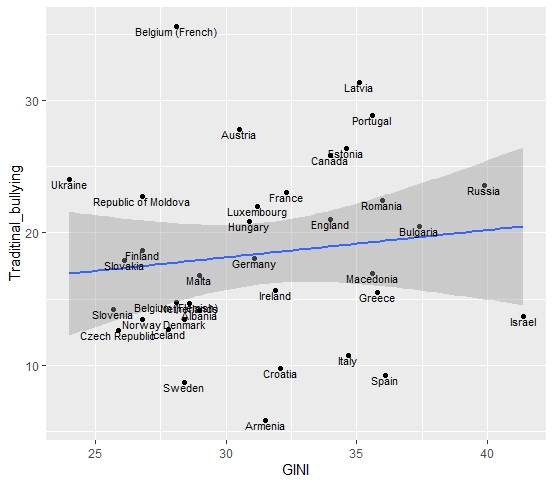

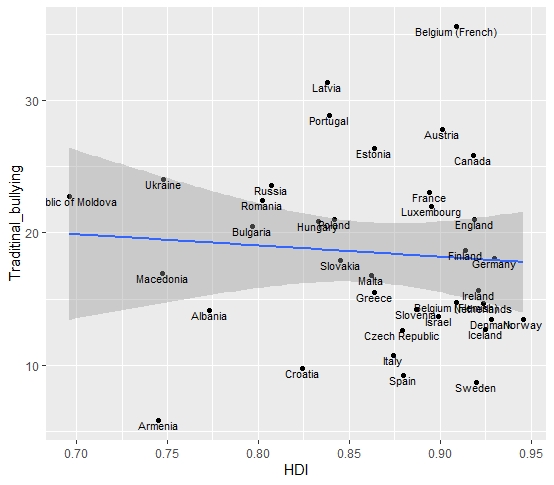
**

**Supplementary Figure 1: Associations of prevalence of traditional bullying victimization with common global indices among adolescents in 40 LMIC-HICs.**

**
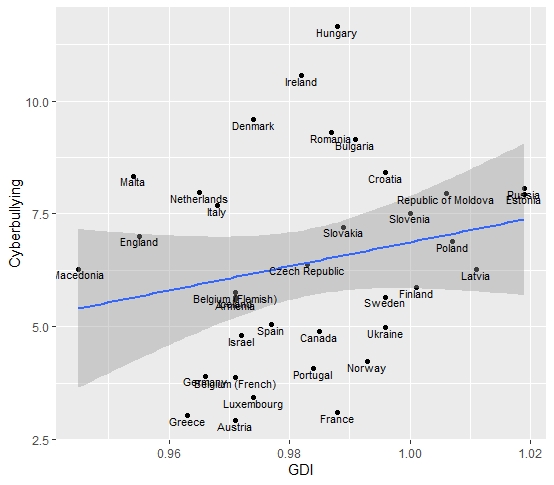
** **
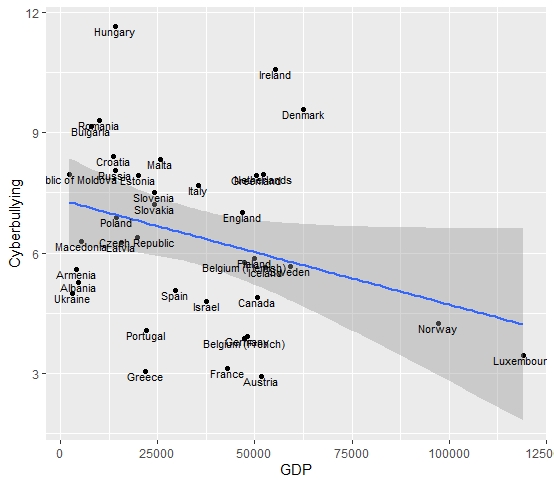
**

**
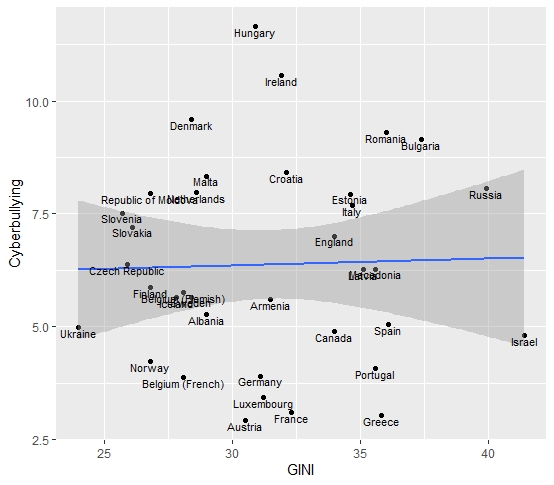
** **
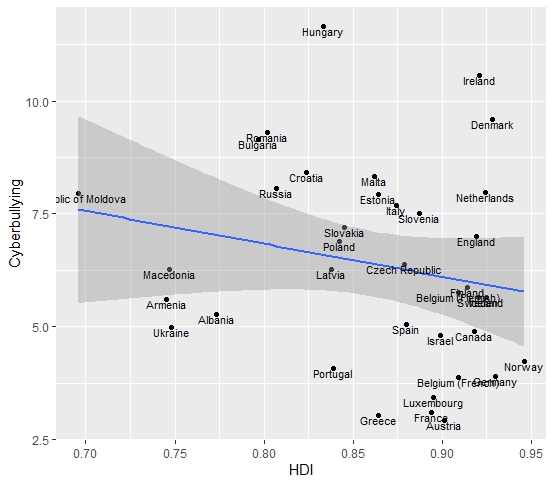
**

**Supplementary Figure 2: Associations of prevalence of cyberbullying victimization with common global indices among adolescents in 40 LMIC-HICs.**

**
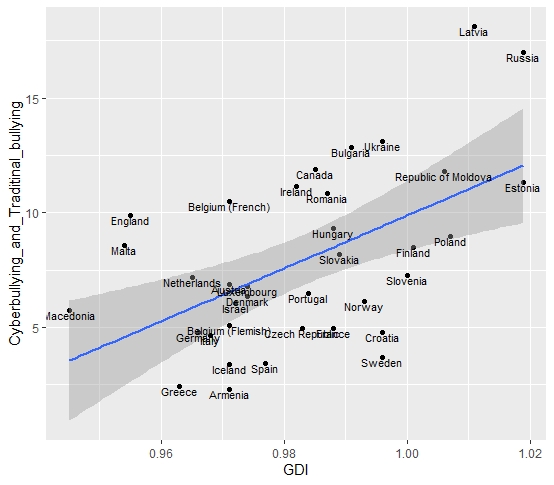
** **
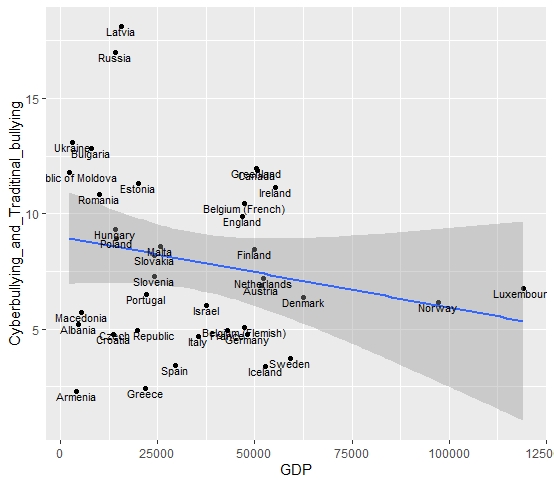
**

**
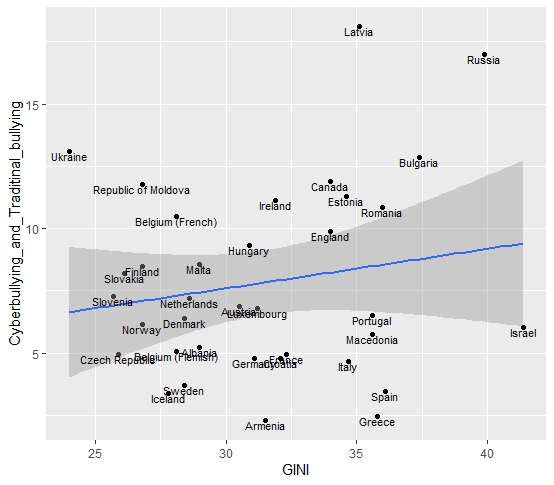
** **
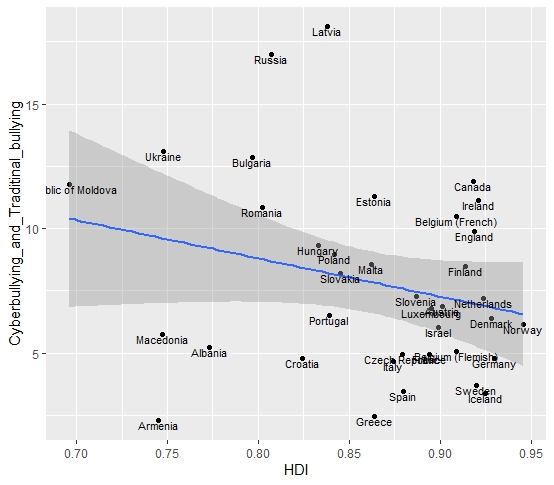
**

**Supplementary Figure 3: Associations of prevalence of Combined traditional and cyberbullying with common global indices among adolescents in 40 LMIC-HICs.**

**Supplementary Table 1: Country specific prevalence of different forms of bullying victimization**

| **Country/WHO Region** | **Traditional bullying victimization** | **Cyberbullying victimization** | **Combined traditional and cyberbullying victimization** |
| --- | --- | --- | --- |
| Austria | 2.17 | 1.52 | 0.41 |
| Germany | 5.8 | 0.99 | 0.51 |
| Hungary | 3.77 | 2.5 | 0.66 |
| Czech Republic | 4.68 | 1.53 | 0.77 |
| Israel | 4.35 | 1.94 | 0.83 |
| Slovakia | 3.48 | 1.36 | 0.94 |
| Finland | 10.24 | 1.04 | 0.98 |
| Poland | 11.04 | 2 | 1.04 |
| France | 8.1 | 1.06 | 1.07 |
| Belgium (Flemish) | 7.35 | 1.48 | 1.09 |
| Greenland | 8.21 | 2.01 | 1.14 |
| Scotland | 3.16 | 3.04 | 1.19 |
| Malta | 6.88 | 1.3 | 1.19 |
| Armenia | 13.07 | 1.39 | 1.21 |
| Netherlands | 4.76 | 1.64 | 1.32 |
| Denmark | 4.86 | 2.52 | 1.36 |
| Luxembourg | 6.62 | 2.87 | 1.41 |
| Albania | 6.73 | 2.15 | 1.41 |
| Spain | 7.97 | 1.99 | 1.47 |
| Russia | 6.52 | 2.02 | 1.62 |
| Estonia | 8.51 | 1.45 | 1.65 |
| Portugal | 9.45 | 2.09 | 1.73 |
| Sweden | 8.19 | 1.97 | 1.82 |
| Latvia | 10.34 | 1.51 | 1.84 |
| Iceland | 5.51 | 3.44 | 1.85 |
| Macedonia | 10.37 | 2.43 | 1.94 |
| Ireland | 6.15 | 3.21 | 2.16 |
| Norway | 9.7 | 2 | 2.18 |
| Greece | 10.2 | 4.02 | 2.25 |
| Belgium (French) | 17.72 | 1.83 | 2.29 |
| Romania | 8 | 1.61 | 2.3 |
| Wales | 10.28 | 2.11 | 2.44 |
| England | 13.93 | 2.83 | 2.51 |
| Slovenia | 11.12 | 3.05 | 2.79 |
| Canada | 11.52 | 2.43 | 3.12 |
| Italy | 19.04 | 2.99 | 3.6 |
| Bulgaria | 11.07 | 4.48 | 3.63 |
| Ukraine | 9.37 | 2.43 | 3.67 |
| Republic of Moldova | 13.44 | 4.9 | 4.22 |
